# Lipid Peroxidation Induces Reactive Astrogliosis by Activating WNT/β-Catenin Pathway in Hydrocephalus

**DOI:** 10.21315/mjms2020.27.3.4

**Published:** 2020-06-30

**Authors:** Wihasto Suryaningtyas, Muhammad Arifin Parenrengi, Abdul Hafid Bajamal, Fedik Abdul Rantam

**Affiliations:** 1 Department of Neurosurgery, Faculty of Medicine Universitas Airlangga, Dr. Soetomo General Hospital, Surabaya, Indonesia; 2 Department of Veterinary Microbiology, Faculty of Veterinary Medicine and Laboratory for Stem Cell Research, Institute of Tropical Disease, Universitas Airlangga, Surabaya, Indonesia

**Keywords:** hydrocephalus, lipid peroxidation, WNT/β-catenin signaling, reactive astrocyte, microglia

## Abstract

**Background:**

Hydrocephalus induces mechanical and biochemical changes in neural cells of the brain. Astrogliosis, as the hallmark of cellular changes in white matter, is involved in demyelination process, re-myelination inhibitory effect, and inhibition of axonal elongation and regeneration. The pathophysiology of this process is not well understood. The purpose of the present study is to elucidate the effect of lipid peroxidation product on astrogliosis through WNT/ β-catenin in kaolin-induced hydrocephalic rats.

**Methods:**

The study used kaolin-induced hydrocephalic rats. Obstructive hydrocephalus was expected to develop within seven days after induction. The hydrocephalus animals were killed at day 7, 14 and 21 after induction. One group of the saline-injected animals was used for sham-treatment.

**Results:**

We demonstrated that the hydrocephalic rats exhibited a high expression of 4-hydroxynonenal (4-HNE) in the periventricular area. The expression of β-catenin also increased, following the pattern of 4-HNE. Reactive astrocyte, expressed by positive glial fibrillary acidic protein (GFAP), was upregulated in an incremental fashion as well as the microglia.

**Conclusion:**

This work suggests that lipid peroxidation product, 4-HNE, activated the WNT/β-catenin pathway, leading to the development of reactive astrocyte and microglia activation in hydrocephalus.

## Introduction

Hydrocephalus is a multifactorial neurologic disorder that can occur in the congenital or acquired setting. Mechanical forces and metabolic changes overlap each other and play a central role in structural and cellular changes. Cerebral blood flow alteration occurs due to the stretching and compression of the capillary vessels in the periventricular area (1, 2). Reduction in cerebral blood flow causes hypoxia, which induces several biochemical cascades including generation of reactive oxygen species (ROS)/reactive nitrate species (RNS), Ca^2+^ influx and generation of reactive hydroxyl molecules. Cell membrane suffers from lipid peroxidation induced by oxidative stress when reactive species level exceeds the antioxidant enzyme level. Some studies revealed the accumulation of lipid peroxidation products, i.e. malondialdehyde (MDA) and 4-hydroxynonenal (4-HNE). Other cellular changes documented in many hydrocephalus studies were astrocyte reaction and microglial activation, together known as astrogliosis. Both these reactions are a normal response of the brain to injury in the acute phase. However, it is attributed to its detrimental effect on structural and functional recovery in a chronic setting in the form of periventricular hypertrophy and proliferation of astrocytes and microglia (3, 4). The chronic reaction may lead to glial scar development that impairs axonal regeneration, inhibits remyelination and causes demyelination (5, 6). Once the reactive astrocytosis is established, shunt placement only temporarily ceases the process and will rise again later, suggesting that surgical treatment fails to completely ameliorate or reverse this reaction in the long run (7).

WNT/β-catenin is a critical conserved signaling pathway involved in the development of multiple organs. It influences the various arrays of the biological process including cell proliferation and differentiation, organ development, tissue homeostasis, stem cell maintenance and pathogenesis of many diseases such as degenerative inflammation and carcinogenesis. Activation of WNT/β-catenin depends mainly on the canonical pathway. A previous study revealed its relation to astroglial response in hydrocephalus. Inhibiting WNT/β-catenin pathway and its gene product cyclin D-1 alleviated reactive astrogliosis that was shown as decreasing glial fibrillary acidic protein (GFAP) (8). Microglia were also affected by WNT/β-catenin in a neurodegenerative study. Specific WNT ligand could increase pro-inflammatory cytokine produced by activated microglia.

The relation between lipid peroxidation and activation of WNT/β-catenin in hydrocephalus has not been studied. We hypothesised that lipid peroxidation is the subsequent reaction of oxidative stress-induced activation of WNT/β-catenin. This event led to the development of astrogliosis.

## Methods

### Animal

Sprague-Dawley rats aged 10 weeks (*n* = 24) were used for the experiment. They were housed in standard cages and provided with a normal 12 h dark/light schedule with free access to food and water. The rats in the hydrocephalus group were sacrificed at 7th, 14th and 21st days and the rats in the sham-treated group were sacrificed on the 21st day after injection.

### Hydrocephalus Induction and Specimen Preparation

Hydrocephalus was induced in 18 rats using percutaneous kaolin suspension injection into the cisterna magna as previously described (9). In brief, anesthesia was accomplished with an intravenous injection of ketamine/xylazine (90/10 mg/kg). The occipital area and the lower neck were shaved and prepared with 70% ethanol and 10% povidone-iodine. We placed the rat’s chest on a 10 cm thick sponge support so that the neck could be flexed to open the foramen magnum. A slow injection of 0.05 mL of 20% kaolin suspension through foramen magnum was employed. All rats were observed until they recovered from anesthesia and then housed in a standard environment. Hydrocephalus was developed within seven days after injection. The clinical diagnosis of hydrocephalus was made by observing its mobility, gait abnormality and the hunched back appearance. The sham-treated rats only received a sterile saline injection at the same place as the hydrocephalus groups. On the designated days, the rats were sacrificed by decapitating the head. The brain was harvested and fixed for 48 h in 4% paraformaldehyde at room temperature.

### Immunohistochemistry Staining

The brain was embedded in a paraffin block and sliced in 5 μm sections. For immunohistochemistry (IHC) staining, the paraffin sections were stained with mouse monoclonal antibody (Santa Cruz Biotechnology, Texas) for detecting the expressions of 4-HNE (1:300 dilution), Ki-67 (1:500 dilution), GFAP (1:500 dilution) and Iba-1 (1:500 dilution) according to the manufacturer’s instructions. Images from slides were viewed at 20× and 40× magnifications under Nikon H600L microscope, camera DS Fi2 and analysed using NIS Elements Basic Research imaging software (Nikon Corp, Japan). The results were scored using Immuno Reactive Score (IRS) as suggested by Remmele and Stegner (10).

### Statistical Analysis

Data were presented as mean ± SD. Statistical analysis for comparison among groups was performed using ANOVA. Statistical significance was considered for a *P*-value of 0.05 or less. The test was performed using statistic software SPSS version 24 (IBM, Armonk, NY, USA). Path analysis using Categorical Regression with optimal scaling was performed to determine the correlation coefficient between variables (beta coefficient).

## Results

### Hydrocephalus Increased 4-HNE Expressions

**Figure 1:**
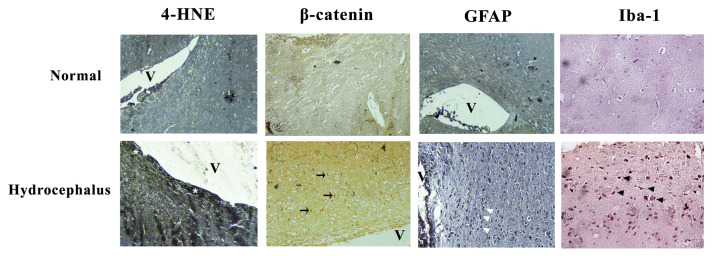
The representative images of 4-HNE, β-catenin, GFAP and Iba-1 expressions in the periventricular white matter of normal and hydrocephalic rats. Expression of 4-HNE (white asterisk) was prominent in hydrocephalic group, especially in periventricular area. Expression of β-catenin (black arrow) was higher in hydrocephalic rat than in normal group. GFAP positive cells (white arrowhead) that represented reactive astrocytes were more abundant in hydrocephalus brain compared to the normal brain. The appearance of Iba-1 positive cells (black arrowhead) showed that the microglia was activated in hydrocephalus group. Activated microglia were barely visible in normal group. All images were taken at 40× magnification except Iba-1 that was taken at 100× magnification

**Figure 2:**
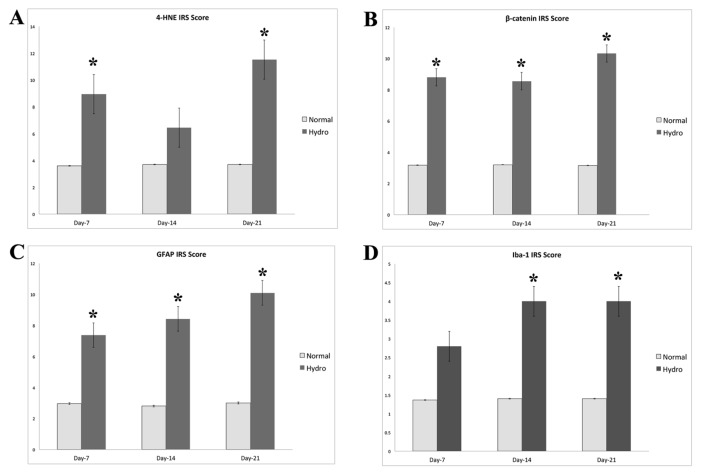
The expressions of (A) 4-HNE, (B) β-catenin, (C) GFAP and (D) Iba-1. All protein expressions were documented as IRS score. The difference between the hydrocephalic and normal group is considered significant when *P* < 0.05 (asterisk)

Immunohistochemical appearance of 4-HNE showed that the lipid peroxidation process was involved in hydrocephalus (Figure 1). The hydrocephalus group demonstrated a significantly higher 4-HNE expression (*P* < 0.001) on day 7 (8.97 ± 2.98) and day 21 (11.52 ± 0.43) than sham-treated group (3.61 ± 0.99) (Figure 2). Immunohistochemistry results showed that the periventricular area, hippocampus, external capsule, and striatum had a higher expression of 4-HNE compared to the cortical area. The coefficient correlation between hydrocephalus and 4-HNE (beta coefficient = −0.878; *P* < 0.001) showed that hydrocephalus was significantly upregulated at 4-HNE level.

### 4-HNE Induced WNT/β-Catenin Activation

We investigated the WNT/β-catenin canonical pathway activation by measuring the cytoplasm β-catenin level (Figure 1). The immunohistochemistry score of β-catenin expression was significantly higher in hydrocephalic rats than in the sham-treated group (*P* < 0.05). Its level followed the fluctuated pattern of 4-HNE. We found β-catenin score increased in day 7 (8.70 ± 0.88), slightly declining in day 14 (8.43 ± 0.42) and increasing further in day 21 (10.41 ± 0.42) (Figure 2). There was no significant difference between day 7 and day 14. The coefficient correlation between 4-HNE and WNT/β-catenin (beta coefficient = −0.843; *P* < 0.001) showed a significant positive correlation between the 4-HNE levels and WNT/ β-catenin. The adjusted R^2^ revealed that 80% of the WNT/β-catenin activator was through lipid peroxidation products. The correlation of increased 4-HNE with WNT/β-catenin activation was measured in path analysis.

### WNT/β-Catenin Activation Stimulated Reactive Astrocytes

Reactive astrocyte was considered as the pathological hallmark in hydrocephalus. The hydrocephalus group showed a significantly higher GFAP expression (*P* < 0.001) than in the normal group (2.96 ± 0.28). In the hydrocephalus group, GFAP expressions increased from day 7 (7.47 ± 0.77) to day 21 (10.21 ± 0.10) (Figure 2). The activation of WNT/β-catenin was correlated significantly with astrocyte reactivity as shown by the positive significant correlation (beta coefficient = −0.987; *P* < 0.001) between WNT/β-catenin and expression of reactive astrocyte.

### Microglial Activation in Hydrocephalic Rats

Increased expression of Iba-1 was found in several areas of the brain, especially at the periventricular area, white matter (external capsule), and striatum after induction of hydrocephalus (Figure 1). The hydrocephalus group had a significantly higher score of Iba-1 at day 14 (4.00 ± 0.90) and day 21 (4.00 ± 0.42) compared to the normal group (1.38 ± 0.60) (*P* < 0.001) (Figure 2). The coefficient correlation between hydrocephalus and microglial activation (beta coefficient = −0.754; *P* < 0.001) showed that hydrocephalus was significantly activated microglia.

## Discussion

Hydrocephalus induces injury to the brain through mechanical and biochemical alterations. Hydrocephalic rat model exhibited a hunched-back appearance, less mobility, gait abnormality, and balance impairment. Their hind limbs moved in a short wide-based step. The progressive ventricular enlargement causes mechanical stress to the periventricular white matter and the cerebral blood vessels in that area (2, 11). The biomechanical injury starts as the blood flow decreases further, triggering the hypoxic-ischemic cascade. The protein-lipid damage and lipid peroxidation at the cellular level occur due to oxidative stress. It produces aldehyde such as malondialdehyde (MDA) and 4-HNE (12, 13). In our study, the expression of 4-HNE increased 3 to 4 times in the hydrocephalic animal. Del Bigio et al. (12) also reported similar evidence but to a lesser degree. On day 14 after induction, the expression of 4-HNE declined temporarily and bounced up on day 21 after kaolin induction (Figure 2). This fluctuating pattern of 4-HNE showed a temporary recovered blood flow in two weeks after the induction or cessation of oxidative process in the early period of hydrocephalus development. Correlating the evidence of hydrocephalus with lipid peroxidation product, 4-HNE, captured the evidence of lipid peroxidation process.

The involvement of 4-HNE in modulating downstream signaling has been reported (14). The association of 4-HNE with WNT/β-catenin signaling was reported in diabetic retinopathy (15). Although WNT/β-catenin signaling modulates various biological and pathological processes, its association with hydrocephalus has not been established. There is only one study by Xu et al. that reported the involvement of WNT/β-catenin in hydrocephalus (8). Our study showed that the high level of 4-HNE in hydrocephalic rats induced WNT/β-catenin. The upregulated 4-HNE level increased the activity of WNT/β-catenin. The correlation was significantly positive with a correlation factor of 0.8. It means 80% of the WNT/β-catenin were through the lipid peroxidation product. Its involvement in the signaling pathway, especially in the canonical WNT pathway, was revealed in this study.

WNTs are secreted cysteine-rich glycoproteins ligands that bind to a complex of frizzled (Fz) receptors and low-density lipoprotein receptor-related protein 5 or 6 (LRP5/6) and regulate target genes expression through an intracellular signaling pathway (16). WNT ligands-LRP6 binding dimerises with the Fz receptor, which is the critical step in the activation of the WNT pathway. In the basal state, β-catenin level, the downstream effector of the canonical WNT/β-catenin pathway is kept low by a 'destruction protein complex' containing glycogen synthase kinase-3β (GSK3β). The WNT-Fz-LRP5/6 complex will inhibit the phosphorylation of β-catenin that results in β-catenin accumulation. β-catenin is then translocated into the nucleus and binds with the T-cell factor to regulate the expression of target genes (16). Regarding the mechanism of WNT/β-catenin pathway activation by 4-HNE, it increases phosphorylation of LRP6, an initial step in WNT/β-catenin pathway activation. However, the exact mechanism remains unclear. One possible explanation is that 4-HNE modifies LRP6 or proteasome and inhibits its degradation. This assumption is supported by the report that HNE inhibits LRP6 degradation rather than increasing its production (15).

Activation of WNT/β-catenin plays a role in astrogliosis reaction in hydrocephalus. Our study showed a similar result that astrogliosis was correlated with the activation of WNT/β-catenin pathway. Currently, there are three theories regarding the origin of reactive astrocytes following brain injury. First, GFAP+ astrocytes come from itself that go through the dedifferentiation process and then divide. Second, it comes from oligodendrocyte progenitors that are found throughout the white matter. Third, it is originated from subventricular zone migrating progenitors. The activation mechanism of reactive astrocyte by WNT/β-catenin involving oligodendrocyte progenitor cell (OPC) is supported by evidence that WNT/β-catenin signaling occurred in GFAP+ cells and progenitor cells. High β-catenin tone inhibited oligodendrocyte progenitor maturation into immature oligodendrocyte. On the other hand, high WNT-3a increased GFAP+ cells (17–19). As the number of mature oligodendrocyte falls, the remyelination process will be altered. Inhibition of WNT/β-catenin signaling in knockout animals reduced the OPC reaction that surrounded the penumbra of the injured area. Suppression of OPC reaction to injury muted the reactions of microglia and astrocytes. The reduction of the GFAP+ reactive astrocyte occurred on 5th day post-injury (20).

Reactive astrocyte has dual appointments based on its appearance period. Its protective action is expected to appear at the early phase as it can eliminate excessive extracellular glutamate and store and supply energy to neighboring cells. Its protective effect will decline after seven days and the neurotoxic phenotype will replace it (21). The hallmark of the neurotoxic state is increased inflammatory chemokine-cytokine, glial scar-forming extracellular matrix and inhibition of synaptogenesis (22, 23). WNT/β-catenin signaling is also related to pathogenic development of glial scar in the brain as well as in different organs, such as lung fibrosis, liver fibrosis, skin fibrosis, and renal fibrosis (24). Detrimental effects of glial scar include inhibition of axonal sprouting (25), remyelination (26), and axonal regeneration as well as production of an extracellular matrix, chondroitin sulfate proteoglycan (CSPG), that are involved in demyelination process (27, 28). Several studies addressed these detrimental effects on neural regeneration and plasticity. Surgical and non-surgical measures were explored to minimise or inhibit the astrogliosis with varying degree of success (29, 30). A surgical approach by shunting resulted in a partial reduction of reactive astrocytosis. The inflammatory phenotype continued to express at the later stage of the chronically shunted animal (31).

Microglia activation in hydrocephalus involved TLR-4 and RAGE as the receptor of Damage associated molecular patterns (DAMPS) and downstream signaling of the nuclear factor-κB (NF-κB) pathway (32). Activated microglia has a modulatory effect in the development of astrocytosis and determines the property of astrocytes under pathological conditions. The neurotoxic reactive astrocyte phenotype is induced by microglia-derived cytokines. Microglia, through NF-κB signaling, potentially amplify the inflammatory reaction by upregulating the level of cytokines and chemokines. Interleukin-1 alpha (IL-1α) and TNF-α are two classic cytokines that induce the neurotoxic type of reactive astrocyte. This phenotype shows a compromised ability in synaptogenesis, promotion of neuronal survival, and release of neurotoxins, which induce apoptosis in neurons and oligodendroglia (22). Reactive astrocyte secretes chemokines: monocyte chemoattractant protein (MCP)-1/ CCL2 and IFN-γ inducible protein (IP)-10/ CXCL10. These chemokines play a role in microglial activation as well as motility. It allows the recruitment of microglia and circulating macrophages to enter the brain parenchyma through a compromised blood-brain barrier. The reactive astrocyte also secreted Lipocalin-2 (Lcn-2), which enhances microglial inflammatory activity (33).

**Figure 3:**
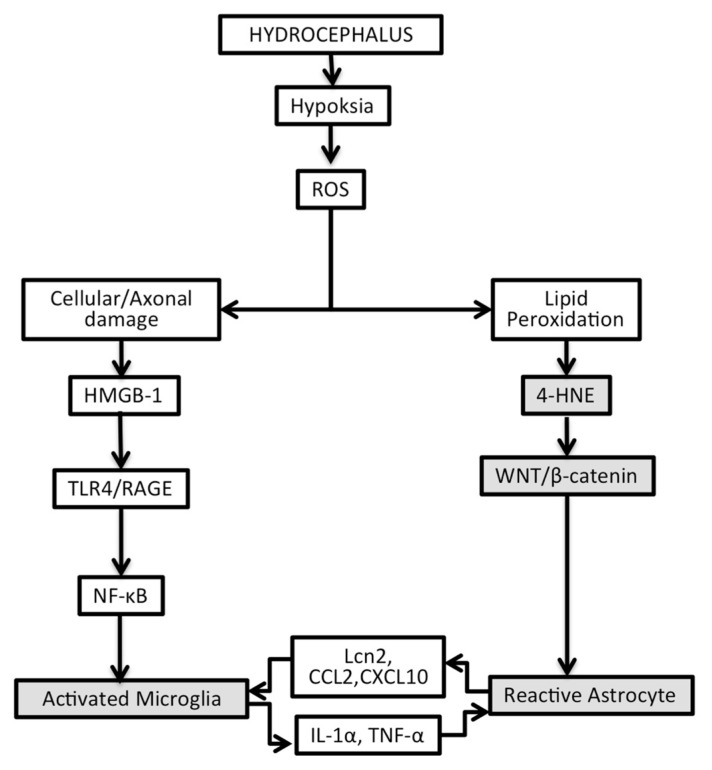
The diagram depicts the pathobiology of astrogliosis as the product of cellular biochemical changes in hydrocephalus

Taken together, the results of our study and the latest theories in hydrocephalic pathology revealed the involvement of hypoxia-induced cascade that eventually activates the microgliaastrocyte crosstalk (Figure 3).

## Conclusion

The hallmarks of cellular changes in hydrocephalus are development of reactive astrocyte and microglial activation. Hypoxia-induced lipid peroxidation activated WNT/β-catenin signaling pathway through its aldehyde products, 4-HNE, which in turn upregulated GFAP+ reactive astrocytes and microglial activation.
